# CRISPR/Cas9 therapeutics: a cure for cancer and other genetic diseases

**DOI:** 10.18632/oncotarget.9646

**Published:** 2016-05-26

**Authors:** Faheem Ahmed Khan, Nuruliarizki Shinta Pandupuspitasari, Huang Chun-Jie, Zhou Ao, Muhammad Jamal, Ali Zohaib, Farhan Ahmed Khan, Muthia Raihana Hakim, Zhang ShuJun

**Affiliations:** ^1^ Key Laboratory of Agricultural Animal Genetics, Breeding and Reproduction, Education Ministry of China, Huazhong Agricultural University, Wuhan, People's Republic of China; ^2^ State Key Laboratory of Agriculture Microbiology, Huazhong Agricultural University, Wuhan, People's Republic of China; ^3^ Key Laboratory of Special Pathogens and Center for Emerging Infectious Diseases, Wuhan Institute of Virology, Chinese Academy of Sciences, Wuhan, China; ^4^ Department of Cardiovascular Medicine, Zulfiqar Ali Bhutto Medical University, Pakistan Institute of Medical Sciences, Islamabad, Pakistan; ^5^ Tongji Medical College, Huazhong University of Science And Technology, Wuhan, China

**Keywords:** CRISPR/Cas9, genetic diseases, cancer, genome editing, next generation sequencing

## Abstract

Cancer is caused by a series of alterations in genome and epigenome mostly resulting in activation of oncogenes or inactivation of cancer suppressor genes. Genetic engineering has become pivotal in the treatment of cancer and other genetic diseases, especially the formerly-niche use of clustered regularly interspaced short palindromic repeats (CRISPR) associated with Cas9. In defining its superior use, we have followed the recent advances that have been made in producing CRISPR/Cas9 as a therapy of choice. We also provide important genetic mutations where CRISPRs can be repurposed to create adaptive immunity to fight carcinomas and edit genetic mutations causing it. Meanwhile, challenges to CRISPR technology are also discussed with emphasis on ability of pathogens to evolve against CRISPRs. We follow the recent developments on the function of CRISPRs with different carriers which can efficiently deliver it to target cells; furthermore, analogous technologies are also discussed along CRISPRs, including zinc-finger nuclease (ZFN) and transcription activator-like effector nucleases (TALENs). Moreover, progress in clinical applications of CRISPR therapeutics is reviewed; in effect, patients can have lower morbidity and/or mortality from the therapeutic method with least possible side-effects.

## INTRODUCTION

In recent years, available therapies for cancers have been evolving to the betterment of prognosis in patients. Chemotherapy, radiotherapy and surgery are used in combination to reduce the cancerous cells to remission, that increases lifespan to a maximum of five years. However, harmful side effects and toxicity increases the mortality whilst it significantly reduces the quality of life [1]. The understanding of cancer biology is of key importance to develop novel anti-cancer therapies. The present day advances in sequencing technology have helped to explore the cancer genome more efficiently with much lower cost. Cancers are characterized by DNA and RNA alterations including mutations, gene duplications and changes in messenger RNAs. The integrative approach to utilize genomic and transcriptomic advances can unveil the complete picture of individual genome. This approach is also being used in clinical setting to make critical decisions regarding patient treatment [2].

Cancer exsists in multiple complex forms making it difficult to prevent and/or treat. It is of ought most importance to study etiology, pathogenesis, prognosis and its phenotypes to develop new therapies and improve the existing treatments. Mutations are among the leading causes of cancers. To date approximately 140 genes with deleterious mutations are reported. This further complicate the ability to develop appropriate effective therapeutics [3]. The multiple steps in cancer development provides ample time for therapeutic strategies to work against its appearance. The initiation of cancer begins with DNA mutation but several contributing factors arise from the epigenome of the individual which needs novel approaches in maintaining the homeostasis. Recent advances in sequencing technologies have helped sequence a diversified variety of cancer neoplasms, providing novel insights into cancer prevention and therapy.

The human genome is composed of two haploid sets of 23 chromosomes, each containing approximately six billion nucleotides. Approximately 20,000 genes exist in each set of chromosome. These genes are transcribed into messenger RNAs (mRNA), ribosomal RNA (rRNA) and transfer RNA (tRNA) constituting the whole transcriptome of an individual [4, 5]. Other species of RNAs termed as non coding RNAs (ncRNAs) which includes micro-RNAs (miRNAs) that does not encode proteins but may activate or inhibit the gene expression [6]. miRNAs have been reported to stimulate several genes and are evolving as important players in therapeutic strategies against several diseases [7]. The completion of Human Genome Project in 2003 established foundations for precision medicine based on sequencing technologies continues its journey from RNAi, ZFNs and TALENs and now it steps into a unique CRISPR/Cas9 genome editing tool. The present day understanding of gene functions and mutations owing to omics results in establishment of various molecular tools to diagnose risk factors for various diseases having genetic components enlisting Diabetes, Alzheimer's, Huntington, Duchenne muscular dystrophy, Inborn blindness and Rheumatoid arthritis. The CRISPR/Cas9 technology has presently been shown to correct the mutations causing those diseases and has a potential to be developed as a promising therapy at genetic level to protect patients at risk.

**Figure 1:**
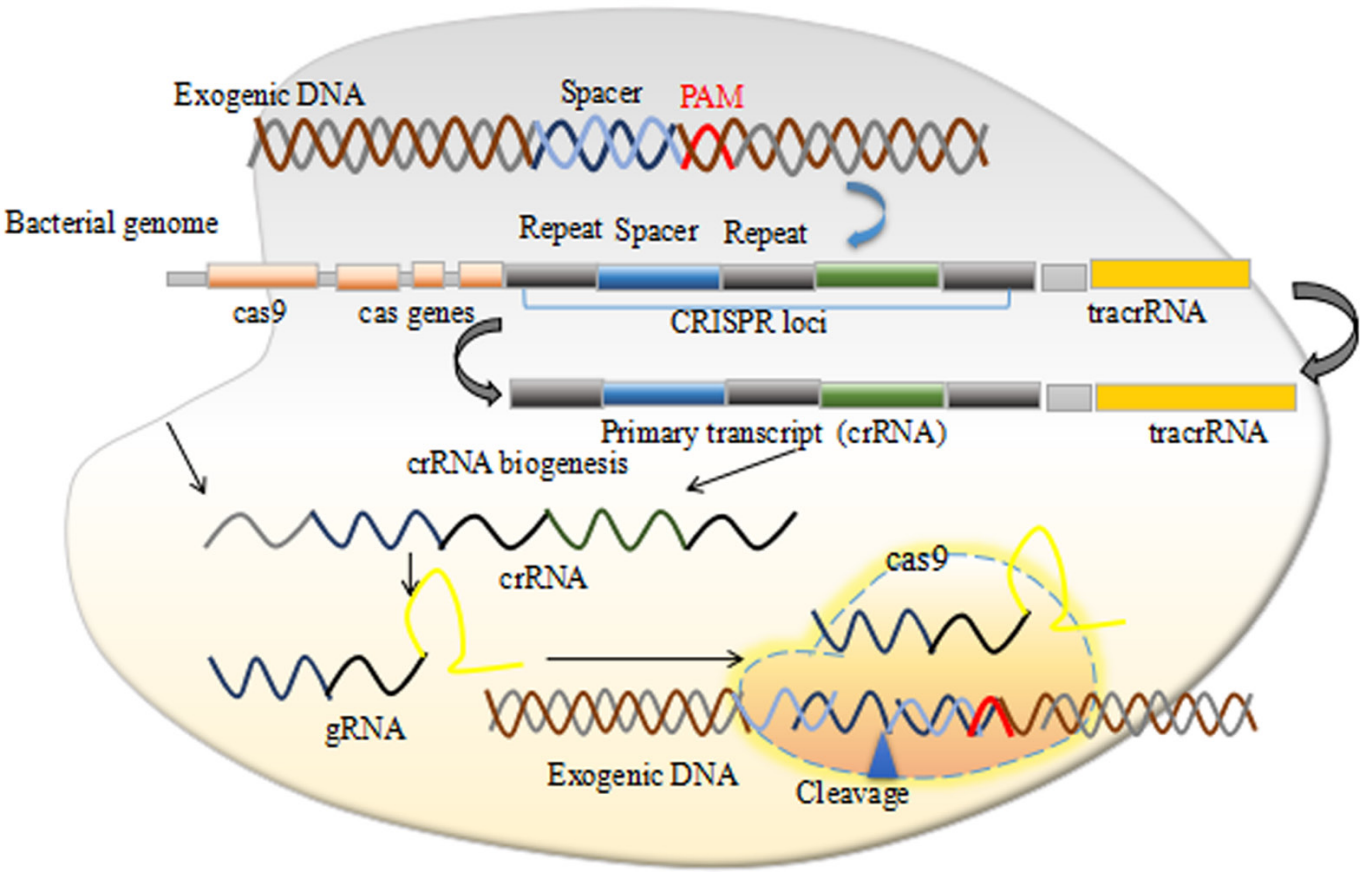
The CRISPR/Cas9 mechanism

Previously, several therapies to treat cancer were introduced but none sustained for long time. Major causes of failure include the development of the self-resistance and the deleterious side effects. Previously DNA domain binding proteins zinc fingers nucleases (ZFNs) and transcription activator-like effector nucleases (TALENs) were employed to treat cancers but their efficiency was limited due to their inability to effectively target the epigenetic changes that occur during carcinogenesis [8]. Recently a more versatile genome editing technology, clustered regularly interspaced short palindromic sequences (CRISPRs) associated with HNH domain protein Cas9 shows promise towards reliable long term cancer therapy. CRISPR/Cas9 is an adaptive immune system in bacteria and archaea against phage invasion in natural environment. Bacteria evolve this system through capturing DNA sequences and used it as a memory to be identified as enemy and destroy it on its attack in future (Figure 1) [9]. This natural adaptive immunity of bacteria and archaea can be redesigned to achieve desired genome editing and more importantly repurpose it as a therapy against long awaiting cancerous and genetic disorders.

Epigenetic changes defines the environment for cancer development. The mutations in tissue development genes stimulate cancer development. Previously Khan and colleagues discussed SUMOylation as one of the epigenetic events causing cancer that might be exploited in novel therapeutic strategies to cure cancers [7]. The present review is an attempt to see the applicability of CRISPR/Cas9 system in cancer and genetic disease therapies. Furthermore, several genetic mutations and suitability to CRISPR/Cas9 system is explored to provide researchers to focus on the translation of laboratory research to clinics.

## NEXT GENERATION SEQUENCING, MUTATIONS, GENETIC DISEASES AND TUMORIGENIC GENES

The understanding of cancer has been revolutionized by the present day next generation sequencing technologies (NGS). The NGS provides the identification of specific mutations relevant to cancers and other genetic diseases at genomic level that can be edited by genome editing technologies the ZFNs, TALENs and CRISPRs or the combination of them. The NGS technologies include whole genome sequencing, whole exome sequencing, RNA sequencing, reduced representation bisulfite sequencing, and chromatin immunoprecipitation sequencing each of which is employed for specific objectives reviewed in Yadav et al., 2015 [10]. In cancers often the whole exome sequencing is performed to get specific mutations at the cellular levels.

A substantial quantity of recent researches identifies mutations in onco-genes that causes cancer. The well known onco-genes are *p53, AKT1* (v-akt murine thymoma viral oncogene), *BRCA1* (breast cancer in females and prostate cancer in males), *BRCA2* (breast cancer in females and prostate cancer in males), *ALK* (anaplastic lymphoma receptor tyrosine kinase), *BRAF* (B-Raf proto-oncogene, serine/threonine kinase), *EGFR* (epidermal growth factor receptor), *KRAS* (Kirsten rat sarcoma viral oncogene), *MET* (proto-oncogene, receptor tyrosine kinase), *NRAS* (neuroblastoma RAS viral (v-ras) oncogene homolog), *RET* (ret proto-oncogene), *ROS1* (ROS proto-oncogene 1, receptor tyrosine kinase), *Bcl11A* (B-cell CLL/lymphoma 11A), *Bcl11B* (B-cell CLL/lymphoma 11B) and *HER2/neu* (erb-b2 receptor tyrosine kinase2). It is a necessity to understand the normal signaling pathways as well as dysfunctional signaling mediated by gene mutations. Some of the mutations in genome causing cancers and other genetic diseases are listed in Table1.

Several studies in past proposed therapies that might be useful in treating cancers. Among those therapies the nuclease guided therapies carries the potential to correct the mutations and dysfunction in a homeostatic epigenetic environment that causes cancers.

The correlation of chronic inflammation is well defined in cancer acceleration but its cellular and molecular mechanisms remain unknown. A recent study in this regards found that Kras^G12D^ an onco-gene that induces expression of IL-17 receptors on pancreatic intraepithelial neoplasia (PanIN) and also synergistically employs T_H_17 and IL-17+/gdT Cells stimulate the expression of PanIN epithelial gene expression hence providing insight into the pancreatic neoplasia [18]. Lung cancer that accounts for 1.6 million deaths worldwide in 2012 [19] have been associated with Rasonco-genes (Hras, Kras, Nras). Recently mutations in Ras genes were shown to dysfunction the wild type allele and hence generating proto-oncogenes that suppresses the carcinogenesis [20]. The findings of To and colleagues is of high importance as it is possible to produce desired mutations in Ras genes in patients at risk of lung and other cancers. The generation of mouse cancer models become efficient with CRISPR/Cas9 technology. Several laboratories have reported useful results in the progress towards cancer cure such as the NANOG and NANOGP8 involvement in malignant potential of prostate cancer [21] which can be corrected with CRISPR/Cas9 or in combination with TALENs or either ZFNs.

Apart from cancers there are several other genetic diseases including Huntington, Alzheimer's, Diabetes, Sickle cell anemia which are caused by mutations in relevant genes. Notably many of these mutations are now known with the help of NGS technologies. The developments in the genome editing technologies have the potential to precisely correct those mutations and revert the defect to its original form at DNA level. The programmed nucleases ZFNs and TALENs were used previously to correct these deleterious mutations, however, the success of the technology fall well short of expectations.

**Table 1:** Cancers, genes, mutations, and CRISPRs editing ability

Cancers/ Genetic diseases	Mutations that can be corrected with CRISPR/Cas9*	CRISPR/Cas9 Gene targets	References
Lung	exon 19 deletion and L858R	EGFR	[11]
Breast	G309A, D769H, D769Y, V777L, P780ins, V842I, and R896C and BRCA1/2 mutations	HER2/Neu, BRCA	[12]
Thyroid	C228T and C250T	TERT promoter	[13]
β- Thalesemia	IVS2-654 (C > T)	HBB	[14]
Huntington	p.(Gln302) and p.(Tyr539Cys)	RNF216	[15]
Limb girdle muscular dystrophies types 2B and 2D	c.5713C>T; p.R1905X, and missense c.229C>T; p.R77C	Dysferlin, and alpha-sarcoglycan	[16]
Alzheimer's	H214N, R220P	Presenilin 1	[17]

* These are only few of the representative mutations causing cancers or other genetic diseases

## GENOME EDITING TOOLS

The interpretation of gene expression, its stimulatory or suppressive role in biological pathways and its interaction with disease phenotypes remains the core aim of classical genetics and today's age molecular biology [22]. The design of any therapeutic technology at molecular level that can cure diseases should have the ability to precisely correct malfunctioned cells and pathways. The development of RNAi technology in the early 90's and its application in mammalian cells to unveil the molecular functions of genes gave rise to the era of reverse genetics. Since the discovery of RNAi technology more efficient tools naming zinc finger nucleases (ZFNs), TALENs and CRISPRs [23, 24] are developed which can perform the genome wide screens efficiently and have recently been employed to correct several environmentally induced mutations and inborn genetic defects.

The modifiable ability of genome editing nucleases to make specific double stranded DNA breaks (DSBs) which are primarily repaired by naturally present non-homologous end joining (NHEJ) DNA repair pathway that is prone to frame-shift mutations resulting in gene disruption [25, 26]. This condition can be precisely corrected by providing a template along with nucleases that will follow a homologous repair (HR) pathway to mend DSB. It has been previously reported that a nuclease induced DSB near a disease mutation can significantly enhance HR pathway [27]. Hence it is very much possible to correct the mutated gene by providing a template of wild type gene thus greatly enhances its applications in biomedical research.

CRISPRs were discovered firstly in *Escherichia coli* where Ishino and colleagues observed unusual repeat structures in 3′ flanking region of *iap* gene [28] with several later observations of similar structures in other bacterial species, and named CRISPR by Jansen and colleagues [29]. It was later known that the CRISPR loci captured spacer sequences from the invading viruses and used them as a memory to provide bacterial host and or archaea to develop an adaptive immunity through the Cas proteins which makes a double stranded DNA breaks (DSBs). Based on the phylogeny of *Cas* gene, crRNA biogenesis and mechanism of nucleic acid cleavage (DNA and RNA), more than 13 different CRISPR-Cas systems have been recognized which is classified into three major groups (I, II and III) and at least 12 subtype (A-F) respectively [30, 31]. The recent studies on Cas proteins demonstrate its utility in initial identification and excision of attacking viral DNA genomes [32]. The RNA guided DNA breaks is elucidated by understanding the crystal structure of SpCas9 and constructing the truncated Cas9 mutant that facilitates its *in vivo* therapeutic application by providing a mechanism of its packaging in size restraint viral vectors [33]. The SpCas9 crystal structure opened several avenues for its practical applications and scientists around the globe start working it out in their labs for large functional screens of their libraries [34]. The problem of CRISPRs/Cas9 off-target effects are widely questioned for its clinical application and hence several strategies have been worked out to keep the CRISPRs/Cas9 on target and is extensively reviewed recently [25].

## ALTERING EPIGENOME WITH CRISPRS/CAS9

Epigenetic modifications consisting of DNA methytlation and histone modifications provides an essential environment for stimulating gene expression that defines their cell proliferation and differentiation activity [35, 36]. Histone proteins responsible for packaging the whole DNA in eukaryotic cells undergo several epigenetic modification including ubiquitination, phosphorylation, SUMOylation, and acetylation. All those are of reversible nature and are under the control of epigenetic modification enzymes [37, 38]. These epigenetic covalent modifications of histones are of high importance in repression or activation of gene expression [39]. Apart from histone modifications, the structure of chromatin is also defined by DNA methylation. The first ever epigenetic modification identified is carried out by DNA methyltransferase enzymes (DNMTs) [8], that provides environment to prevent binding of transcription factors and/ or bring repressive protein complexes to DNA [40]. DNA methylation is more stable in comparison with post translational modifications of histone but can still be demethylated by active and passive mechanisms, and is responsible for the normal development and cellular differentiation [41]. These days several non coding RNA (ncRNA) species for example micro RNA (miRNA), short interfering RNA (siRNA) are found to inhibit or activate genes that are involved in the epigenetic regulation of critical biological processes of growth and development [42]. miRNAs are implicated in several disease conditions and can be used to target specific gene expression for its up and/or down regulation as required [7]. Recently, CRISPR/Cas9 has been successfully employed to edit genetic switches [9], and many of the miRNAs that are involved in cancer progression and development can be specifically targeted with CRISPR/Cas9 genome editing system.

The recent advances in genome editing technologies especially CRISPR/Cas9 marvelous outcomes have given strong hope to deal with deadly genetic and cancerous diseases. The suppression of gene expression was demonstrated when dCas9 (double mutant) fused with a repressor Krüppel-associated box KRAB, but its genome wide specificity and heterochromatin specificity was not known until reports of its binding HS2 enhancer, having distinct role in expressing many globin genes. The observation of highly specific H3K9 trimethylation and limited chromatin accessibility of enhancer and promoter suggesting individual enhancers can be successfully modified to control epigenome changes [43].

Epigenetic drugs are in extensive use with reliable efficacy but have some strong side-effects that need an alternative platform to specifically modify the epigenome for treating cancers. Epigenetic drugs have been implicated in severe off-target effects that trigger several genes including *p53*, *Akt*, *cMyc* and cause dysfunction in several biological pathways including metabolic and immunity pathways. Hilton and colleagues have recently demonstrated that a fused nuclease-null dCas9 with catalytic core of human acetyltransferase p300 cotransfected with multiple gRNAs in HEK297T cells to target *IL1RN, MYOD* and *OCT4* endogenous promoters resulted in highly specific gene expression through acetylation of H3 lysine27 (H3K27) by its promoters and enhancers, thus provides robust tool for gene modifications [45]. As the major portion of human cancers is because of the loss in global methylation patterns or hypermethylation of specific loci, the present progress and further findings of epigenome editing tools will be of key interest in curtailing cancers.

## CARRIERS OF CRISPR/CAS9 TO CELLS

There are difficulties in uniform and sustained transportation of CRISPR system to cells, that needs to be addressed. The specialized methods developed to deliver Cas9 and gRNA to target site within a cell or tissue claims to aid CRISPR specificity. The nuclease genome editing technologies effectively and efficiently alter genome sequences, hence providing an opportunity to correct disease causing mutations, but requires potent delivery methods to cells. The Cas9 can be delivered to cells as mRNA, however the mRNA is unstable and is not suitable for long term gene therapy purposes, however the alterations to genome remains and widely used in model organisms including mouse, zebrafish, *Drosophila* and *C. elegans* [46, 47]. The expression of Cas9 and gRNA complex in cultured mammalian cells is mostly done physically by delivering non-replicating plasmids expressing these cassettes. These methods involve electroporation [48], microinjection [49, 50] lipofection [51, 52] nucleofection [51–53]. The main disadvantage of using plasmid is the random integration of plasmid or its part in both off-target and on-target sites leading to insertion inactivation of genes. To address these shortcomings, several efforts were put in to deliver Cas9 protein in conjugation with cell penetrating proteins (CPPs) complexed with guide RNA (gRNA) that forms nanoparticles with positive charge showed efficient disruption of genes [54]. An enhanced delivery vehicle inspired from DNA nanoclews and DNA nanoparticles is recently been reported having the capability of simultaneously delivering the Cas9 protein and sgRNA to human cell nuclei and disrupt genes efficiently while maintaining cell viability [55]. Viral vectors such as Adeno-associated virus (AAV), lenti-virus are broadly used as gene delivering tool due to efficient introduction of an exogenous DNA fragment into genome by robust HDR. Although low pathogenicity and oncogenic risk makes AAV a suitable choice, yet large size of SpCas9 (4.2 kb in length, ~1,400 amino acids) limit AAV transducing abilities as it allows only 4.7 kb fragment to be transduced. This problem has been resolved by a smaller size ‘SaCas9’ (3.3 kb). AAV mediated delivery of this Cas9 variant shows the same efficiencies as the natural SpCas9 on the same genomic loci with no off-target mutation [56].

**Figure 2:**
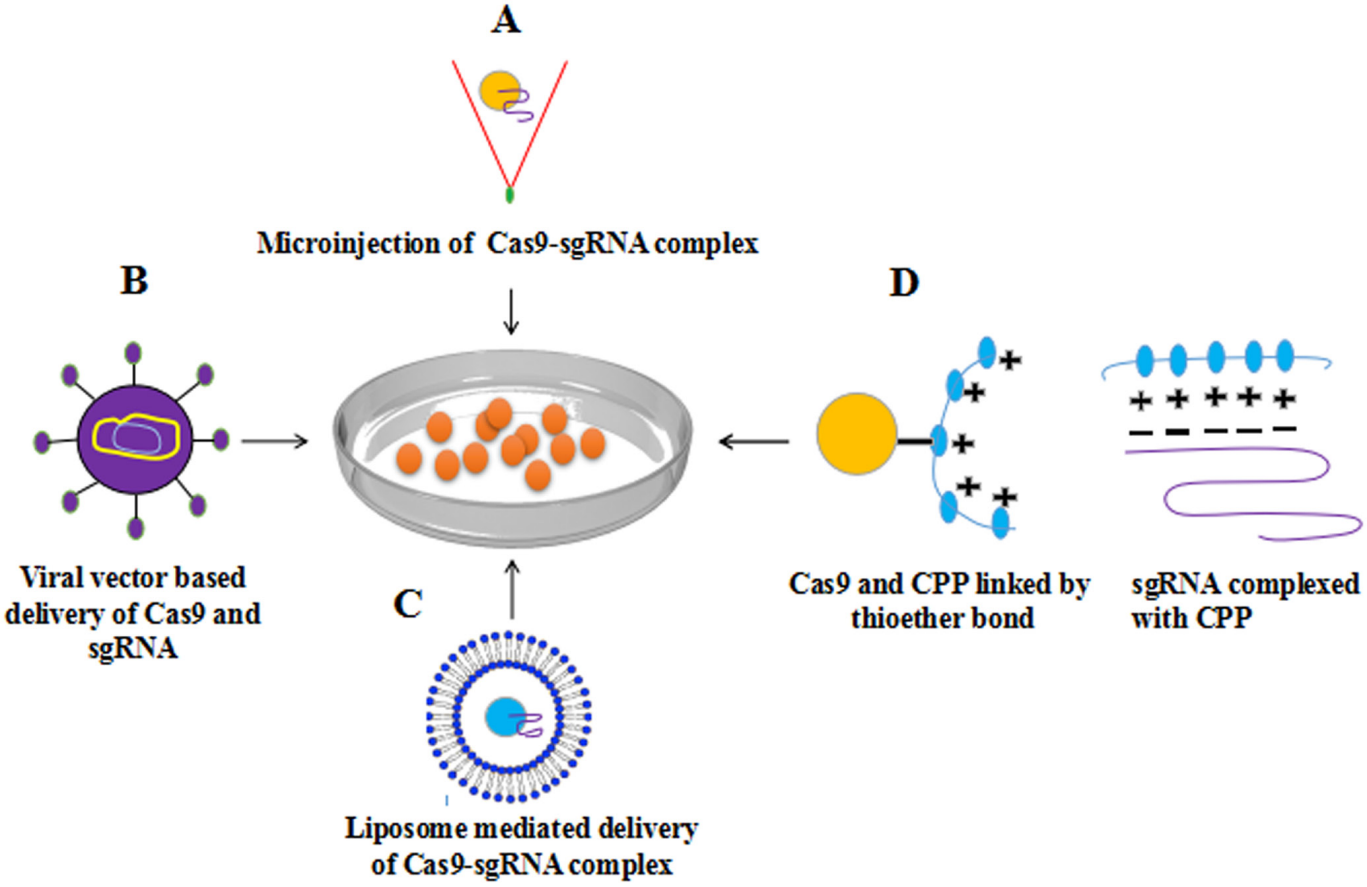
Methods for delivery of Cas9-sgRNA complex to cell **A.** Microinjection based delivery of Cas9-sgRNA **B.** viral vector (AAV) based delivery **C.** Lipofection **D.** Cell penetrating peptides (CPP) based delivery of Cas9-sgRNA complex into mammalian cells have shown successful genome editing with high efficiency.

Mostly for gene therapy *ex vivo* treatment of cells is performed which is later transferred to body and the most stable way of transferring Cas9 and gRNA in this regard is with the use of non-viral DNA plasmids [46]. It is of paramount importance to develop tools and methods to efficiently and specifically stimulating or inhibiting gene expression to achieve desired results for cancer therapy. The strategy to control cancer disease can be done in two ways either by removing tumor tissue or to control transgene expression by the tumor-specific promoter such as telomerase promoters which are often involved in cancers. The *in vivo* delivery of nucleases is addressed by Zuris and colleagues, they conclusively show that proteins delivery by cationic lipid is a viable approach for genome editing and is capable of carrying Cre recombinase, TALE, CRISPR/Cas9: gRNA and Cas9 transcription factor activators [57] (Figure 2).

The delivery of ribonucleoprotein (RNP) (Cas protein and gRNA) to cells induced site specific mutation of up to 79% and reduced off-target cleavage associated with plasmid transfection at off-target sites that differ by one or two nucleotides from on-target sites [58, 59]. The efficiency of delivering RNPs *via* electroporation transformation is reported two times higher as compared to plasmid mediated transformation. Through RGEN RNP delivery, two endogenous genes (*dpy-3 and unc-1*) in *C.elegans* have been heritably knocked down without indel formation [60]. This method has some advantages as this complex provides a control Cas9 amount to the cell followed by rapid degradation. For therapeutic application the invention of safe and improved delivery method of Cas9-sgRNA into the cell or organisms is urgently needed. Improvement of existent method and invention of new method as a cargo such as use of nano particles might enable efficient and specific genome editing [58, 61].

## CRISPRS PATH TO CLINICS

The rapid development of the genome editing technologies need an adequate attention towards improving pre-clinical and clinical assays to assess the toxicity, off-target effects, and other possible side effects. Several attempts have been made in using CRISPRs to correct the mutated genes. One of such study was carried out in mice cancer model with mutated *Pten* and *p53* genes. The mice were transfected with a vector carrying designated CRISPR through a tail vein to achieve 20% of hepatocytes to transform through blood that also successfully corrected *β-catenin* gene mutation frequently involved in cancer with CRISPR [62].

Burkitt's lymphoma is a cancer caused by mutations in *cMyc* gene and almost every patient suffering from it, has Epstein-Barr virus (EBV) infection. Recently CRISPR/Cas9 system is used against EBV that reduced the viral load and tumor proliferation [63]. Furthermore, recent studies demonstrates successful editing of tumor suppressor gene *Trp53* in *Arf−/−Eμ*-Myc lymphomas [64], the over-expression of Myc gene is responsible for several kind of lymphoma cancers [7]. Subsequent studies shows *Mll3* as another important tumor suppressor gene disruption *via* CRISPRs *ex vivo* in acute myeloid leukemia [65]. The successful application of the genome editing tool TALENs in one year old girl patient of leukemia provides the grounds for the use of more efficient CRISPRs application in clinics.

**Figure 3:**
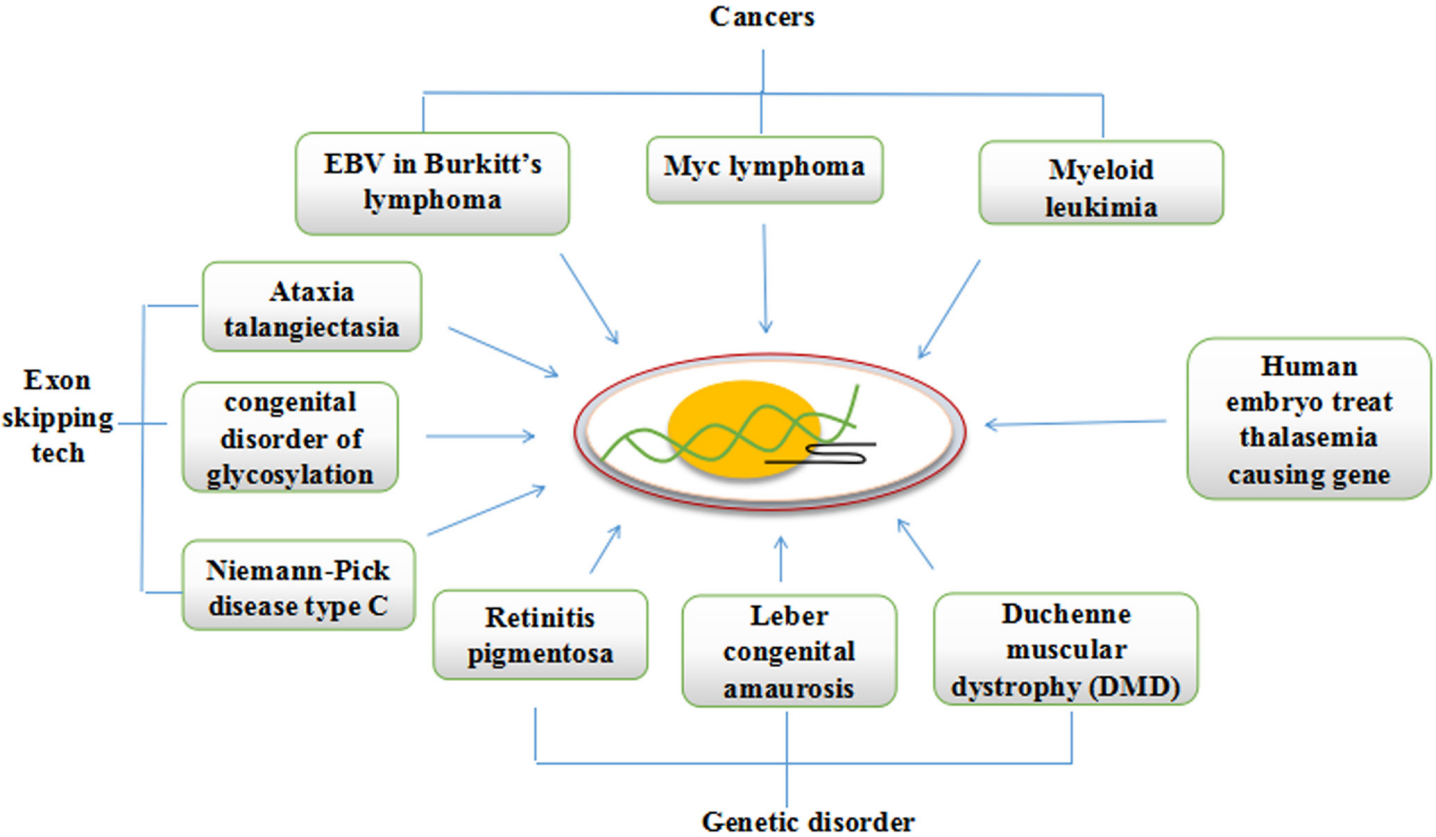
CRISPR/Cas9 can be redesigned to cure mutations causing cancers and genetic diseases

Apart from cancers, CRISPRs are also used to correct several genetic diseases. An inherited eye disease Retinitis pigmentosa that causes breakdown of photoreceptor cells resulting in gradual loss of vision is been recently edited successfully in iPSC for *RPGR* gene, which in health state is responsible for production of proteins involved in normal vision, to give new hope to patients blinded with Retinitis pigmentosa [66]. Efforts are put in Editas pharmaceuticals to make CRISPR therapeutics against children genetic disease Leber congenital amaurosis, that causes blindness by editing the defected gene with CRISPR in eye cells and plans to launch it by the end of the year 2017. Another recessive X-linked disease the Duchenne muscular dystrophy (DMD), is primarily caused by a frame-shift mutation in dystrophin protein which is essential for proper functioning of muscles, and is very much suitable for genome editing inspite of its very large size of 79 exons. It does not require whole of the gene to express and with little changes to sequence which causes disease can bring improvements in muscle functioning. Thus an exon skipping technology can be applied that is proved successful in mouse model of Duchenne muscular dystrophy [67–69]. The exon skipping technology with CRISPR/Cas9 opens the door to treat several other diseases such as Ataxia telangiectasia, congenital disorder of Glycosylation, and Niemann-Pick disease type C caused by errors in splicing. A well renowned experiment in SUN-YATSEN university China in human embryos to treat Thalasemia causing gene in human embryos shows only a few embryos out of 80 received a corrected form of gene copy (Figure 3). These examples of successful clinical application with CRISPR defines bright future of the technology, but have still to work out several preclinical and clinical assays to determine the side effects on patients health, the immunogenic responses to vector carriers, and possible drawbacks on over all genome as a result off-targets. The studies in mouse models confirmed the tumor suppressor activity of *SWI/SNF* subunits. A recent study demonstrates that mutations in EZH2 affects the tumor suppressor activity of *SWI/SNF* subunits and it also suggests that inhibitors of EZH2 in developmental phase will also not fully control the oncogenic activity of EZH2. Hence carefully designed CRISPR/Cas9 system can be utilized to correct the mutations that can help regain the tumor suppressor activity of *SWI/SNF* subunits [70].

## CHALLENGES IN CRISPR/CAS9 CLINICAL THERAPEUTICS

**Figure 4:**
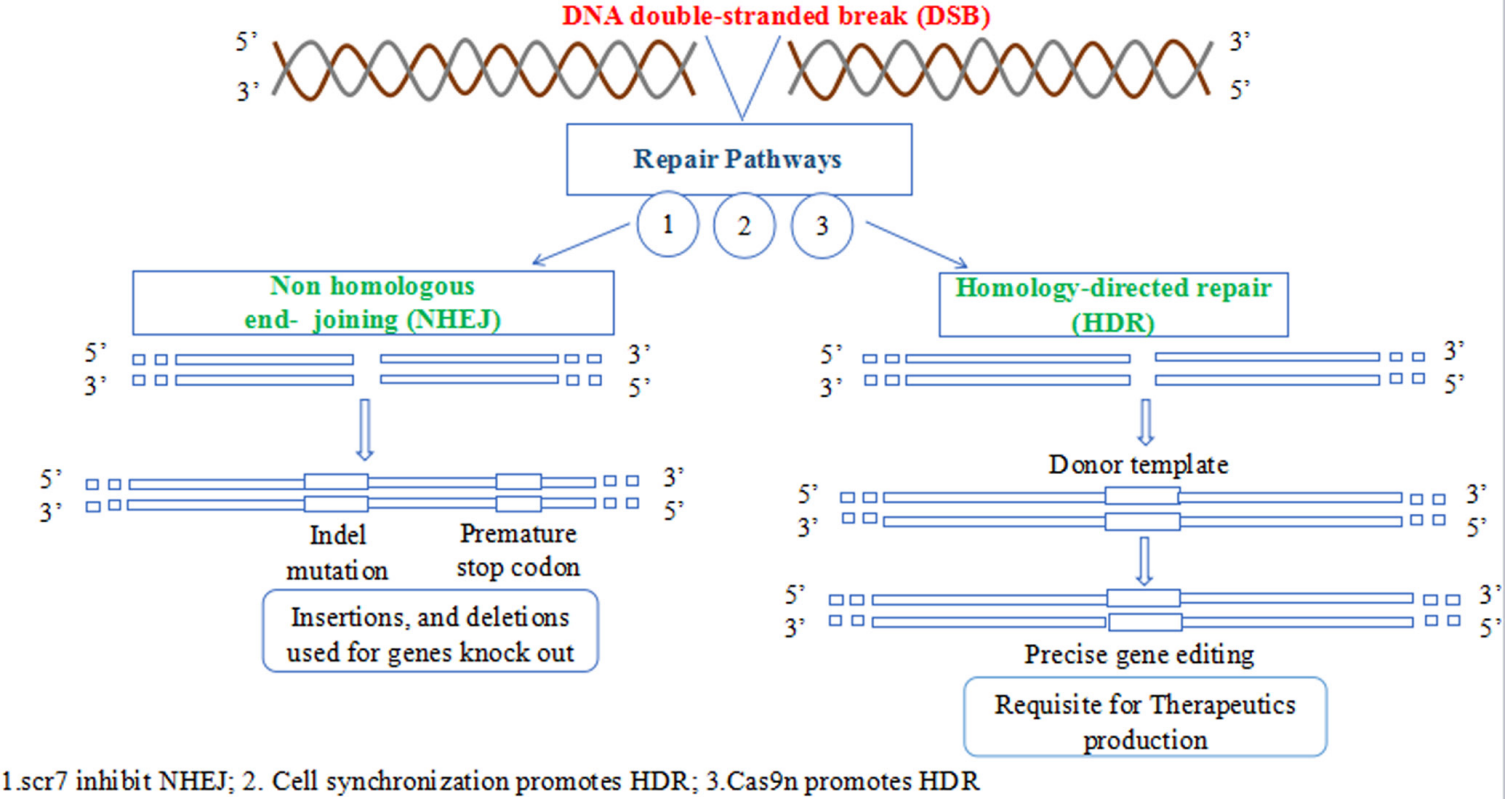
The two DNA repair pathways NHEJ is naturally favored while HDR pathway has therapeutic application in correcting several mutated genes.

There are some obstacles that limits the commercial therapeutic application of CRISPR. The gene editing ability of Cas9 is of paramount importance. The dsDNA break by Cas9 is followed by two natural pathways present in the cell; Non-homologous end joining (NHEJ) and homology directed repair (HDR). NHEJ is naturally favored pathway for gene correction in nature but is error prone and causes undesired mutations hence is not suitable for the application of CRISPR as therapeutic agents. HDR on the other hand is accurate and error free but is not naturally favored pathway for DSBs correction, hence requires means to make HDR to be favored over NHEJ in natural environment to efficiently translate CRISPRs benefits to clinics (Figure 4). NHEJ is mostly dominating during G1, S and G2 phase while HDR in late S and G2 phase [71]. These two pathways have been manipulated by researchers for genome editing using CRISPR in mammalian cell for the first time [52, 53]. The editing frequency achieved by NHEJ ranges from 2% to 25%, which cause high efficiency deletion of the intervening sequence [52]. HDR pathway uses donor DNA as a template to repair the DSB in a ‘copy-and-paste’-type using homologous recombination mechanism. By providing an appropriately designed donor DNA (≥400bp in case of plasmid and 25-65 bp in case of single stranded oligodeoxynucleotides) [72], precise small or large modifications can be made to the genome (HR-mediated genome editing). The genome editing ability of CRISPR-Cas9 using HDR pathway is not fully developed. As the NHEJ pathway is erroneous causing InDels (insertion-deletion) formation at the cleaved site, may lead to frame shift mutation resulting in a malfunction proteins or non-sense mediated decays of transcripts ultimately causing gene disruption [73]. The rate of specificity increase with increase in HDR mediated repair of DSB induced by CRISPR Cas9. Hence the use of HDR is more favored. However, NHEJ competes with HDR to rectify the DSBs. The use of Scr7 inhibitor antagonistic of DNA ligase IV (a principal enzyme involved in NHEJ repair pathway) increased the genome editing efficiency up to 19 fold by preventing NHEJ [74]. It has been shown that cell cycle synchronization of the nuclease in G2 increases HDR efficiency while reducing unwanted NHEJ events [75]. Similarly substituting the normal Cas9 for Cas9 nickase (Cas9n) activates HDR with low off-target potentials [49]. However the efficiency of recombination is low (1 in 10^6^-10^9^ cells), limiting the large-scale applications of HDR in gene-targeting assay [72]. The specificity and accuracy of the gene editing process following site-specific genomic breaking by RGENs depends upon the nature of donor DNA [76]. If the foreign DNA have a homology more than 400bp with the target, it will lead to more efficient introduction of precise nucleotide substitutions or deletions, endogenous gene labeling, as well as targeted transgene [77]. Increasing the length of homology arms of the homology-directed repair template facilitates targeting efficiency, while increasing the length of the DNA insert reduced it [59].

The specificity of the CRISPRs is also under question and many clinical laboratories are concerned about its off-target effects and the ways that can minimize those off-targets and develop clinical assays to measure. Several advances are made in delivering nucleases to destination cells *ex vivo* and *in vivo* but there is still need to improve the delivery systems for realizing the dream of CRISPRs therapeutics. Besides the specificity, key to success is the isolation of mutant cells (having DNA of interest) from a diverse population of cells.

The evolution of host-pathogen interaction is a continuous process. The natural development of CRISPRs in bacteria and archaea as adaptive immune responses took hundreds of years to fight the invasion of pathogens. Recently some groups identified anti-CRISPRs proteins in viruses that can destroy the bacterial CRISPRs with all its memory records of invading viruses and hence exposing it to the larger threat of attacking viruses. Such findings stress the need to further develop more accurate clinical assays of longer efficacy for the introduced therapeutic CRISPRs against evolutionary pressure asserted by pathogens.

## CONCLUSIONS

There is much buzz around genome editing technologies specially CRISPRs to be used against several life threatening diseases at the molecular level. The phrase “Nip the evil in the bud” may rightly be used for CRISPRs therapeutics but it is critical to develop all the requisite clinical tests for its efficacy, safety and specificity before its use in clinics. The short journey of CRISPRs till now is highly fascinating and provides a significant hope of medical cure against deadly diseases.
